# Identification of New Genes and Loci Associated With Bone Mineral Density Based on Mendelian Randomization

**DOI:** 10.3389/fgene.2021.728563

**Published:** 2021-09-08

**Authors:** Yijun Liu, Guang Jin, Xue Wang, Ying Dong, Fupeng Ding

**Affiliations:** ^1^Department of Orthopedics, The First Hospital of Jilin University, Changchun, China; ^2^Department of Anesthesiology, The First Hospital of Jilin University, Changchun, China; ^3^The Third Department of Radiotherapy, Jilin Provincial Tumor Hospital, Changchun, China

**Keywords:** BMD, GWAS, eQTL, causative gene, disease susceptibility, SMR

## Abstract

Bone mineral density (BMD) is a complex and highly hereditary trait that can lead to osteoporotic fractures. It is estimated that BMD is mainly affected by genetic factors (about 85%). BMD has been reported to be associated with both common and rare variants, and numerous loci related to BMD have been identified by genome-wide association studies (GWAS). We systematically integrated expression quantitative trait loci (eQTL) data with GWAS summary statistical data. We mainly focused on the loci, which can affect gene expression, so Summary data-based Mendelian randomization (SMR) analysis was implemented to investigate new genes and loci associated with BMD. We identified 12,477 single-nucleotide polymorphisms (SNPs) regulating 564 genes, which are associated with BMD. The genetic mechanism we detected could make a contribution in the density of BMD in individuals and play an important role in understanding the pathophysiology of cataclasis.

## Introduction

Bone mineral density (BMD), is a main risk factor for osteoporosis (OP) or systemic bone loss, which is associated with the increasing risk of fragility fracture, especially for older women (Glüer et al., 2004; Cauley et al., 2007). BMD also plays a role for causing bone fractures, including pressure fractures (Nattiv, 2000). Generally, BMD can be detected by dual-energy X-ray absorptiometry (DXA), which is a non-invasive bone densitometry method but hard to implement. Another method to measure BMD is quantitative ultrasound of the calcaneus (QUS), which is flexible, inexpensive, and easier to perform. BMDs at the spine and hip are reported to be highly heritable (Arden et al., 1996; Lee et al., 2006), which could be detected by DXA (Gonnelli et al., 2005), and are fracture risk related to fracture risk (Bauer et al., 2007).

Based on genome-wide association studies (GWAS) analysis using heel ultrasound parameters, Moayyeri et al. (2014) identified mutations at nine loci, including seven previously reported loci. GWAS, so far, have detected more than 100 genetic variants associated with BMD, including many significant loci associated with risk of fractures. In recent years, more and more BMD risk variants with low frequencies have been detected based on deep whole-genome sequencing. However, most experiment-verified variants can rarely explain approximately 5.8% of the phenotypic variance in BMD (Zheng et al., 2015). Estrada et al. (2012) identified 62 significant SNPs by performing a meta-analysis consisting of 17 BMD GWAS studies, which focused on lumbar spine or femur neck. Kemp et al. (2017) performed a genome-wide association screen by UK Biobank and identified 307 independent SNPs located in the 203 loci. However, it remains elusive on how these genetic loci lead risk to BMD based on linkage disequilibrium phenomenon (LD) between detected SNPs and real causative mutations. In addition, due to the strict statistical significance threshold set in GWAS analysis, it is difficult to detect co-pathogenic loci in a single GWAS study. Therefore, we need to use other omics data to reveal the potential effect of these weak GWAS association signals on BMD, which may help to understand the heritability of this trait.

By these biological experiments, researchers have found several genes, which are related to BMD. Some researchers have used computational method to identify more BMD-related genes (Wu et al., 2021). Machine learning and deep learning methods have been widely used in the prediction of trait-related genetic factors (Zhuang et al., 2019; Tarwadi et al., 2020; Zhao et al., 2020a). Most of these methods predict the associations between biomolecules by feature extraction and building mathematical models (Tianyi et al., 2021; Zhao et al., 2020b,2021a). However, these studies fail to explain the biological mechanism of results. Therefore, it is necessary to further reveal the mechanism of significant SNPs identified by GWAS (Zhao et al., 2019).

Considering the influence of LD, systematical approaches are proposed to explore the latent regulatory functions of the risk variants reported in previous GWAS studies by integrating multiple omics data (Peng and Zhao, 2020; Zhao et al., 2020c). Since gene expression is an important factor related to genetic mutations and traits, many researchers tried to reveal pathogenesis by gene expression (Zhao et al., 2021b). Researches have detected numerous expression quantitative trait loci (eQTLs) associated with BMD based on eQTL data from primary bone cell cultures (Grundberg et al., 2009; Kwan et al., 2009). Kwan et al. (2009) has found that rs136564 plays an important role in regulating the expression of a novel transcript of FAM118A, and rs136564 is also reported to be related to BMD based on GWAS analysis. Therefore, many studies focused on confirming whether an SNP can be detected by both GWAS and eQTL analysis (Farber, 2012). However, most studies focused on separately analyzing GWAS data and eQTL data rather than in an integrative way to identify disease genes (Farber and Lusis, 2008).

Mendelian randomization approach is proposed as a method of using genetic variants as instrumental variables to examine the causal influence of a modifiable exposure on diseases. Based on this assumption, we can identify the most functionally related genes to diseases. Apparently, complex traits, such as BMD, are not only derived from the effect of a single gene but also the integrated influence from complex biological networks (Schadt, 2009). In this study, we applied the Mendelian randomization (MR) method based on summary statistic data to identify novel causative genes associated with BMD. We first collected two GWAS datasets from UK Biobank [including 394,929 individuals (Zheng et al., 2015)], UK10K [including 32,965 individuals (Kim, 2018)], and blood eQTL data (Westra et al., 2013). Then SMR was implemented to investigate new genes and loci associated with BMD. As a result, we identified 12,477 SNPs regulating 564 genes, which have causal effect on BMD. Finally, we assessed the functional interactions between these genes to examine their underlying functional mechanism.

## Data and Methods

### Data

#### Genome-Wide Association Studies Summary Data

The GWAS summary data were obtained from UK Biobank and UK10K project, respectively. Individuals (394,929) with genotype and phenotype data were collected from the UK Biobank. The DNA variants were filtered by MAF > 0.1%. The dataset from UK10K is composed of 2,882 whole-genome sequencing (WGS data), 3,549 whole-exome sequencing (WES data), 26,543 deep imputation of genotyped samples, and 20,271 *de novo* replication genotyping. The detailed description information of GWAS datasets can be accessed from previous studies (Westra et al., 2013; Zheng et al., 2015).

#### Expression Quantitative Trait Loci Summary Data

It has been validated that bone metabolism is related to various types of cells such as peripheral blood monocyte cell (PBMC), B and T lymphocytes (Chalmers et al., 1981). PBMC plays an important role in studying gene expression functions related to human osteoporosis risk (Liu et al., 2005). They can also be considered as precursors of osteoclasts (Geissmann et al., 2010) and express various cytokines, which are essential in the biological process of osteoclast (Deng et al., 2011). B lymphocytes can also express biological factors associated with osteoclastogenesis and plays an important role in the immune system (Manabe et al., 2001). Recently, studies based on eQTL-mapping methods indicated that most of the disease-causative mutations actually have an influence on the expression level of nearby genes due to the phenomenon of LD (Dubois et al., 2010; Nicolae et al., 2010). Researchers have also identified that *trans*-eQTLs can reveal the downstream consequences of the variants (Fehrmann et al., 2011; Innocenti et al., 2011; Grundberg et al., 2012). In this study, we collected eQTL summary data of 5,311 samples in peripheral blood tissue, which is derived from a total of nine datasets from seven different cohorts (Westra et al., 2013).

### Methods

#### Genome-Wide Association Studies Meta-Analysis

Since GWAS analysis focus on the effect of a single genetic variant, it ignores the interactions between different loci. However, the effect size of an SNP is different from diverse datasets. Thus, we performed a GWAS meta-analysis on two GWAS summary datasets in order to correct the effect size of multiple GWAS datasets. By assigning different weights to each SNP from different datasets, we can integrate these GWAS datasets into a more comprehensive one. There are three measurements to assess the association score between variants and the trait in GWAS dataset, *β*, SE, and *p*-value. *β* measures the estimate of a causative effect between SNP and trait, and SE indicates the standard deviation (SD) of *β*. The *p*-value denotes the significance level of association between SNP and the trait.

Since SE can represent the reliability of *β*, it can be inferred that the bigger the SE, the more inaccurate the *β*. Because SE is the SD of *β*, the weight of *β* can be denoted as the inverse ratio of the SE square. Thus, the weight *w*_*i*_ of β_*i*_ in the *i*th GWAS dataset can be denoted as:

(1) $$w_{i}=1/S\,E_{i}^{2}$$

where *SE*_*i*_ denotes the SD of the SNP in the *i*th dataset.

Thus, we can integrate the effect size measurement *β* between different datasets, and it can be denoted as:

(2) $$\beta =\sum_{i}\beta_{i}\,w_{i}/\sum_{i}w_{i}$$

In the meantime, SE after the integration of the datasets can be denoted as:

(3) $$\text{SE}=\sqrt{1/\sum_{i}w_{i}}$$

Then we calculated the Z-score of SNPs based on the effect size β and SE to obtain the significance of SNPs. Z-score can be denoted as:

(4) Z=β/SE

Then we obtained the *p*-value of the association after the integration of the effect of SNPs from different datasets based on the hypothesis testing of the normal distribution of the Z-score.

Thus, we can integrate multiple GWAS datasets by applying the above method. It can be deduced that the reliability of SNPs and SE are negatively correlated, and the weight of β is lower compared with other datasets, while the SE value is bigger. Thus, the value of β can be corrected across multiple datasets according to different weights.

#### Summary Data-Based Mendelian Randomization Analysis

Multiple potential and unmeasurable confounding factors may lead to huge challenges in inferring the causative relationship between genes and complex traits. However, genetic mutation is a major factor of heredity. Thus, exploring the underlying mechanism of genetic variants is important to reveal the pathologies of complex traits. Due to the linkage disequilibrium, the effect size between SNPs detected by GWAS analysis and BMD may not be accurate. Moreover, GWAS cannot fully explain the association between BMD and SNPs. Thus, the MR method is first proposed to consider a genetic variant as a factor to assess and examine for the effect size of an exposure variable on an outcome (Smith and Ebrahim, 2008). Based on the MR theory, if we use *z* to denote an SNP, *x* as the gene expression, and *y* as the BMD, then the association of gene expression (*x*) and BMD (*y*) can be denoted as *b*_*xy*_,

(5) $$b_{x\,y}=b_{z\,y}/b_{z\,x}$$

where *b*_*zy*_ indicates the association between SNP and BMD, and it can be represented as the slope of z to y. *b*_*zx*_ denotes the association between SNP and gene expression, and it can be denoted as the slope of z to x. *b*_*zy*_ and *b*_*zx*_ can be obtained from two independent GWAS dataset and eQTL dataset.

Then the sampling variance of the estimate value of *b*_*xy*_ can be denoted as:

(6) $$v\,a\,r\,\left({\hat{b}}_{x\,y}\right)=\left[v\,a\,r\,\left(y\right)\,\left(1-P_{x\,y}^{2}\right)\right]\,/\,\left[n\,\,\mathrm{var}\,\left(x\right)\,P_{z\,x}^{2}\right]$$

where *n* denotes the size of samples, ${\hat{b}}_{x\,y}$ denotes the estimate value of *b*_*xy*_ $P_{x\,y}^{2}$ indicates the proportion of variance in BMD, which is explained by gene expression, $P_{z\,x}^{2}$ indicates the proportion of variance in gene expression level explained by SNP. Therefore, the statistic *T*_*SMR*_ is utilized to test the significance of *b*_*xy*_, and *T*_*SMR*_ can be represented as:

(7) $$T_{S\,M\,R}={\hat{b}}_{x\,y}^{2}\,/\,\mathrm{var}\,\left({\hat{b}}_{x\,y}\right)$$

However, it is not realistic, so far, to collect genotype data and gene expression data from a very large sample size. Also, because the effect size of eQTL was unavailable, *b*_*zx*_ can be estimated from the Z-score of eQTL data as ${\hat{b}}_{z\,x}$:

(8) $${\hat{b}}_{z\,x}=Z_{z\,x}\,S_{z\,x}$$

where $S_{z\,x}=1\,\sqrt{2\,f\,\left(1-f\right)\,\left(n+Z_{z\,x}^{2}\right)}$, *f* is the allele frequency, and *n* is the sample size. An unbiased estimate of *b*_*zx*_ could be denoted as ${\hat{\varepsilon }}_{z\,x}$. We therefore have:

(9) $${\hat{b}}_{x\,y}={\hat{b}}_{z\,y}\,/\,{\hat{\varepsilon }}_{z\,x}$$

where ${\hat{b}}_{z\,y}$ denotes the estimate of the effect of an SNP from GWAS data for BMD, and ${\hat{\varepsilon }}_{z\,x}$ is the estimate of the effect of an SNP on the gene expression level from an eQTL data. The Delta method can be utilized to calculate the sampling variance of ${\hat{b}}_{x\,y}$ approximately (Lynch and Walsh, 1998):

(10) $$v\,a\,r\,\left({\hat{b}}_{x\,y}\right)\approx \frac{b_{z\,y}^{2}}{\varepsilon_{z\,x}^{2}}\,\left[\frac{v\,a\,r\,\left({\hat{\varepsilon }}_{z\,x}\right)}{\varepsilon_{z\,x}^{2}}+\frac{v\,a\,r\,\left({\hat{b}}_{z\,y}\right)}{{\text{b}}_{z\,x}^{2}}-\frac{2\,\mathrm{cov}\,\left({\hat{\varepsilon }}_{z\,x},{\hat{b}}_{z\,y}\right)}{\varepsilon_{z\,x}\,b_{z\,y}}\right]$$

where $\text{cov}\,\left({\hat{\varepsilon }}_{z\,x},{\hat{b}}_{z\,y}\right)$ is 0 when ε_*zx*_ and *b*_*zy*_ are derived from independent GWAS and eQTL datasets. Because the distribution of the Z-score is known, while the distributions of ε_*zx*_ and *b*_*zy*_ are unknown, *T*_*SMR*_ can be approximately denoted as:

(11) $$T_{S\,M\,R}=\frac{b_{x\,y}^{2}}{\mathrm{var}\,\left({\hat{b}}_{x\,y}\right)}\approx \frac{Z_{z\,y}^{2}\,Z_{z\,x}^{2}}{Z_{z\,y}^{2}+Z_{z\,x}^{2}}$$

where *Z*_*zy*_ and *Z*_*zx*_ denotes the Z-score derived from GWAS and eQTL data. Since the distribution of *T*_*SMR*_ is **x*^2^ = 1*, the significance of *b*_*xy*_ can be calculated by performing a χ^2^-test of *T*_*SMR*_, which is also the significance of the association between gene and BMD.

#### SMR Analysis for Bone Mineral Density With Expression Quantitative Trait Loci Data From Blood Tissue

We first integrated two independent GWAS datasets to obtain a more comprehensive GWAS dataset. After obtaining β and SE of each SNP based on GWAS dataset and eQTL dataset, respectively, we obtained the estimate of effect size of SNPs on BMD based on integrated GWAS summary data and estimate of effect size on gene expression based on SNPs from eQTL data. We obtained two Z-scores of the same SNP based on two datasets, *Z*_*GWAS*_ and *Z*_*eQTL*_. The GWAS dataset provides the SNPs associated to BMD, and the eQTL dataset provides the association between these SNPs and expression level of gene. Then the SMR method is utilized to examine the effect size of SNPs on BMD excluding some irrelevant factors.

**FIGURE 1:**
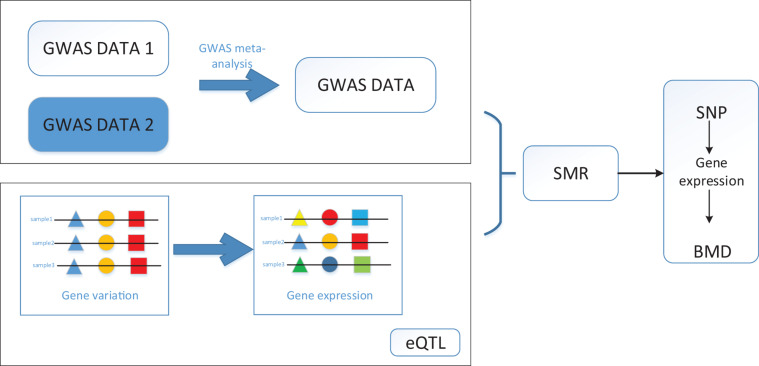
Workflow of SMR on bone mineral density (BMD) based on genome-wide association studies (GWAS), and expression quantitative trait loci (eQTL) datasets.

Since one single SNP can regulate multiple genes, we then identify the causative genes, which are regulated by these SNPs and are associated with BMD. We performed a Bonferroni test to filter the SNPs we obtained for the SMR method. After all, we identified 12,477 SNPs, and 564 genes regulated by these SNPs are associated with BMD. It is clear from the result that most of the causative genes are regulated by multiple SNPs, which means detecting the disease-related genes merely depending on GWAS datasets is not reliable. The workflow is shown in Figure 1.

## Results

**FIGURE 2:**
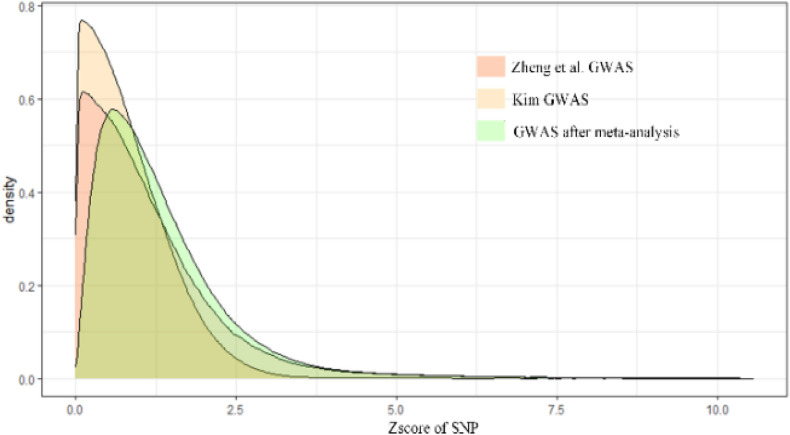
The result of GWAS meta-analysis on BMD.

**FIGURE 3:**
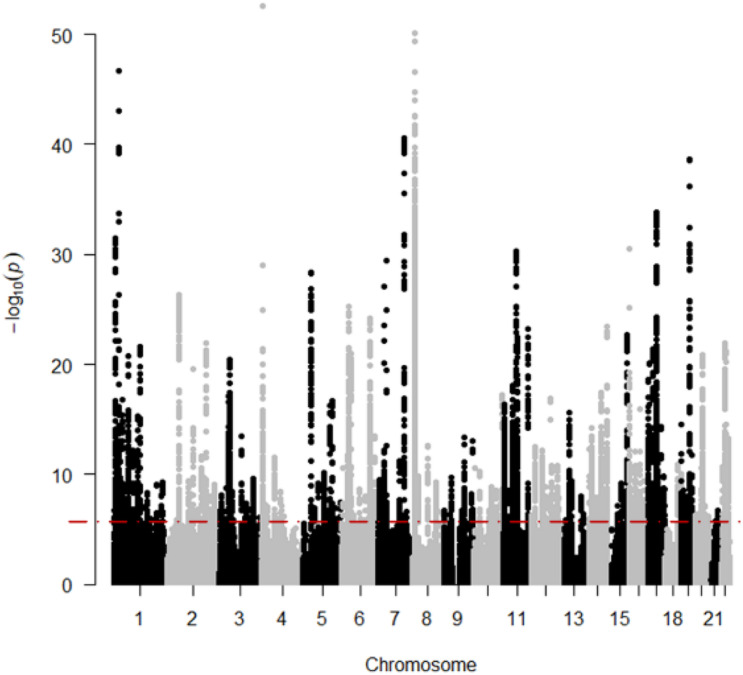
The results of BMD-related genes based on SMR. The red line means “Significant threshold”.

The result of the GWAS meta-analysis is shown in Figure 2. It is apparent that the original datasets from former studies are not consistent. After integration, we obtained a more precise GWAS dataset for BMD. Since there are many overlapping SNPs in the GWAS dataset and eQTL dataset, we have to filter these SNPs to find out whether the genes regulated by these SNPs are associated with BMD. Thus, the SMR method is utilized to examine latent associations between gene expression and BMD. The results of BMD-related genes based on GWAS and eQTL to test for the integrated data are shown in Figure 3. We identified, in total, 12,477 SNPs regulating 564 genes associated with BMD. This indicates that multiple SNPs may cooperate and effect the expression of a single gene. For example, gene FDFT1 is regulated by 451 SNPs, and most SNPs can regulate multiple genes as well, such as rs10085549, rs1073, and so on. They can regulate seven genes. Supplementary Material indicates the significant genes and SNPs related to BMD.

### Case Study

As a result of the SMR method, we identified 12,477 significant SNPs and 564 significant genes associated with BMD. Several significant genes of the results have been reported in recent studies. In the study of Kim (2008), they identified that gene DGKQ is associated with heel BMD. In the study of Wang et al. (2015) they have found the association between FDFT1 and the therapeutic response among Chinese postmenopausal women suffering from osteopenia or osteoporosis. Cdc42 is identified to be strongly related to bone deterioration in experimental osteoarthritis according to the study of Hu et al. (2018). LRP3, TMUB2 has also been reported as a risk factor for BMD of the lumbar spine (LS-BMD) (Zhu et al., 2016). RERE is reported to be a novel suspective gene associated with BMD from a group of Caucasian-origin families (Zhang et al., 2009). In total, there are 10 out of the top 20 significant genes in our results that have been reported to be related with BMD according to previous studies. Table 1 shows these 10 genes and related GWAS studies published previously.

**TABLE 1:** Ten of the top 20 significant genes and related study.

Gene	PubMed ID
DGKQ	PMID:30048462
FDFT1	PMID:25223561
Cdc42	PMID:29314205
LRP3	PMID:27019110
TMUB2	PMID:27019110
ASB16	PMID:32269995
RERE	PMID:18597038
MS4A6A	PMID:33604283
EPDR1	PMID:32619791
SPTBN1	PMID:19801982

### Gene Interaction Network Based on Bone Mineral Density

**FIGURE 4:**
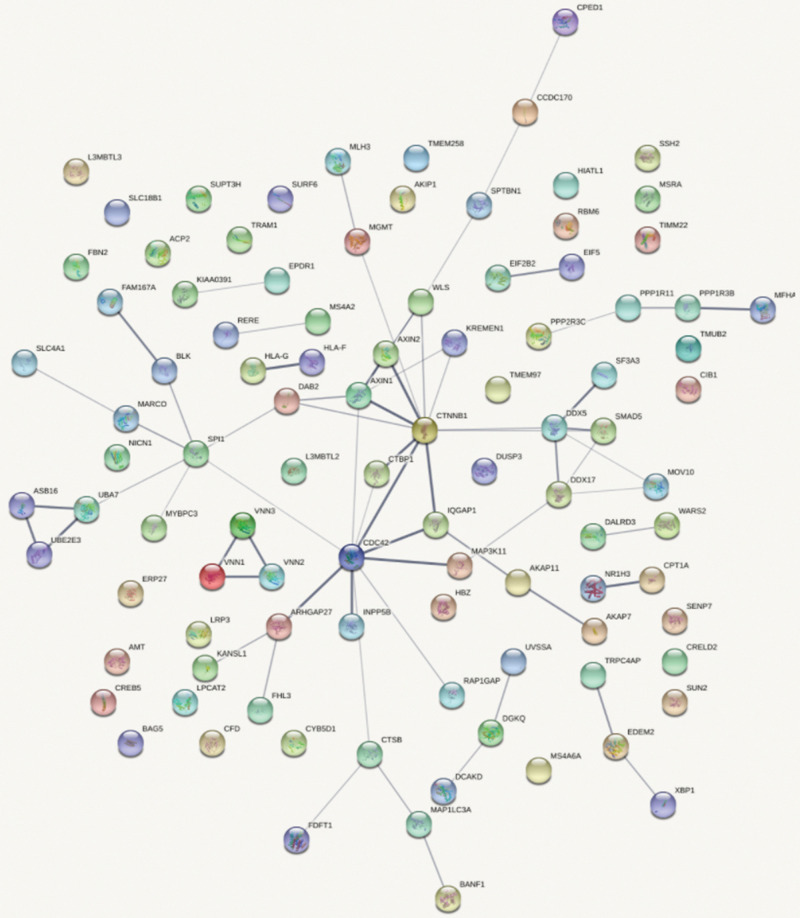
Gene interaction network obtained from the top 100 genes.

**FIGURE 5:**
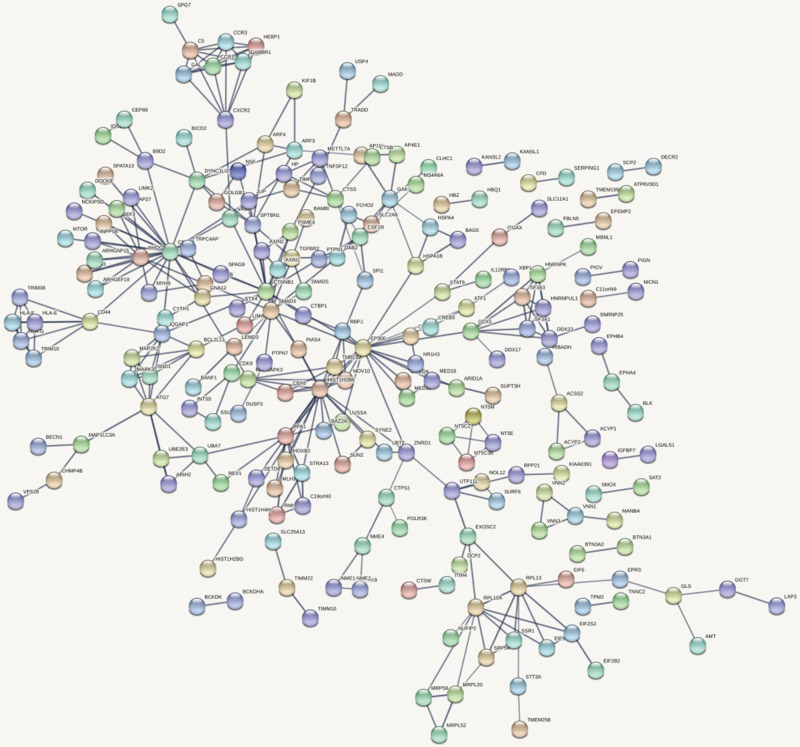
Gene interaction network obtained from all significant genes.

Figure 4 shows the top 100 gene interaction networks derived from the results of the SMR method on BMD. Figure 5 shows the gene interaction network from all significant genes derived from the SMR method. Based on the top 100 gene interaction networks, Cdc42 and CTNNB1 are intensively interacted and significantly associated with BMD. It is known that the process of bone (re)modeling is based on the distinct actions of osteoclasts and osteoblasts, which are achieved by the organization of osteoclast cytoskeleton. Cdc42 belongs to the Rho GTPase subfamily, which is considered to be major regulators of cytoskeleton, and it has been reported to be a prospective therapeutic target for preventing osteoporosis (Ito et al., 2010). CTNNB1 has been reported to be related to BMD in the spine and hips (Estrada et al., 2012).

In total, we identified 12,477 SNPs and 564 genes related to BMD by the SMR method. Then we performed the case study of the identified genes to prove the effectiveness of our BMD-related gene identification method based on multiple omics data integration.

## Conclusion

We use the SMR method to integrate omics data to identify BMD–gene associations. First, we integrated two independent GWAS data sets by adjusting the weights of SNPs to overcome that different GWAS datasets have different sample sizes. Then we reduced the impact of linkage disequilibrium and identified the impact of SNPs on BMD based on GWAS data and eQTL data. Through the Bonferroni test, we obtained 12,477 SNPs and 564 genes significantly related to BMD. Among these genes, 10 of the top 20 risk genes have been previously reported to be associated with BMD, which proves the validity of our method and the correctness of the results, but further biological experiments are needed to verify our results. Our results indicate that BMD is a highly inherited polygenic trait and is significantly associated with osteoporosis. These findings help us reveal the pathology of osteoporosis and determine the relevant pathways and therapeutic drugs.

## Data Availability Statement

The datasets presented in this study can be found in online repositories. The names of the repository/repositories and accession number(s) can be found in the article/Supplementary Material.

## Ethics Statement

Ethical review and approval was not required for the study on human participants in accordance with the Local Legislation and Institutional Requirements. Written informed consent for participation was not required for this study in accordance with the National Legislation and the Institutional Requirements.

## Author Contributions

YL, GJ, and XW wrote the manuscript and did the experiments. FD provided ideas of this work. YL, GJ, and YD analyzed the data. All authors approved the submitted version.

## Conflict of Interest

The authors declare that the research was conducted in the absence of any commercial or financial relationships that could be construed as a potential conflict of interest.

## Publisher’s Note

All claims expressed in this article are solely those of the authors and do not necessarily represent those of their affiliated organizations, or those of the publisher, the editors and the reviewers. Any product that may be evaluated in this article, or claim that may be made by its manufacturer, is not guaranteed or endorsed by the publisher.

## Supplementary Material

Supplementary Table 1 Predicted genes.

Supplementary Table 2 Predicted SNPs.
